# Engineering Tropism of *Pseudomonas putida* toward Target Surfaces through Ectopic Display of Recombinant Nanobodies

**DOI:** 10.1021/acssynbio.1c00227

**Published:** 2021-08-02

**Authors:** Sofía Fraile, María Briones, Mónica Revenga-Parra, Víctor de Lorenzo, Encarnación Lorenzo, Esteban Martínez-García

**Affiliations:** † Systems Biology Department, Centro Nacional de Biotecnología (CNB-CSIC), Campus de Cantoblanco, 28049 Madrid, Spain; ‡ Departamento de Química Analítica y Análisis Instrumental, Universidad Autónoma de Madrid, Campus de Cantoblanco, 28049 Madrid, Spain

**Keywords:** surface display, intimin, nanobody, *Pseudomonas putida*, quartz crystal microbalance, surface attachment

## Abstract

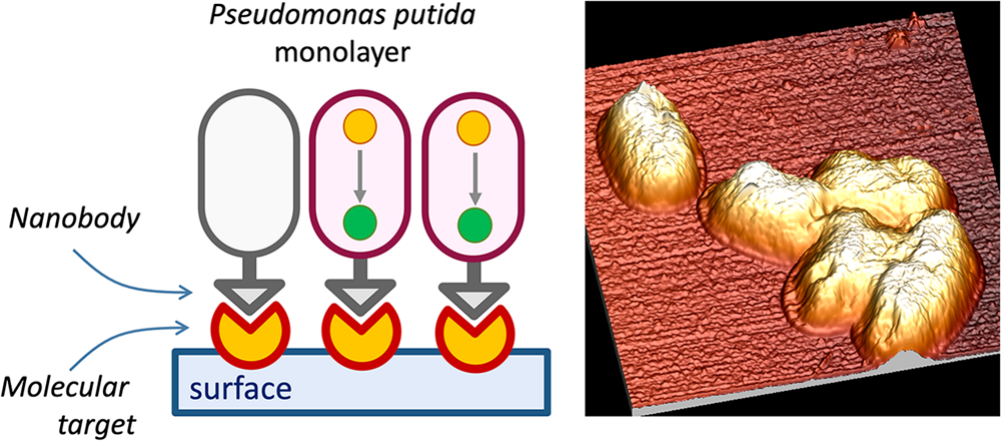

Gram-negative bacteria are endowed with complex outer membrane (OM) structures that allow them to both interact with other organisms and attach to different physical structures. However, the design of reliable bacterial coatings of solid surfaces is still a considerable challenge. In this work, we report that ectopic expression of a fibrinogen-specific nanobody on the envelope of *Pseudomonas putida* cells enables controllable formation of a bacterial monolayer strongly bound to an antigen-coated support. To this end, either the wild type or a surface-naked derivative of *P. putida* was engineered to express a hybrid between the β-barrel of an intimin-type autotransporter inserted in the outer membrane and a nanobody (V_HH_) moiety that targets fibrinogen as its cognate interaction partner. The functionality of the thereby presented V_HH_ and the strength of the resulting cell attachment to a solid surface covered with the cognate antigen were tested and parametrized with Quartz Crystal Microbalance technology. The results not only demonstrated the value of using bacteria with reduced OM complexity for efficient display of artificial adhesins, but also the potential of this approach to engineer specific bacterial coverings of predetermined target surfaces.

Physical interactions of Gram-negative bacteria with both solid surfaces and other organisms are largely mediated by the whole of the specialized structures displayed on the outer membrane (OM), toward which they are targeted upon secretion through a plethora of export systems.^1^ The molecular specimens involved in cellular motion and adhesion, *e.g.*, pili, flagella, fimbriae, extracellular polymeric substance (EPS), *etc*., determine attachment of bacteria toward either other cells or abiotic materials.^2,3^ The soil bacterium and plant root colonizer *Pseudomonas putida*(4,5) is no exception, and such OM-anchored structures are necessary for survival in natural settings where finding the right interaction partners is of essence for endurance under harsh environmental conditions. *P. putida* KT2440 is a nonpathogenic variant^6^ endowed with a metabolic architecture that secures high levels of NADPH to withstand oxidative stress^7^ and possessing also a high tolerance to organic solvents.^8^ A plethora of genetic tools have become available along the past decade to perform virtually any type of genetic modification in this species.^9^ All of these attributes make *P. putida* KT2440 an excellent framework for biotechnological applications required for the production of high value products, specially under harsh physicochemical conditions.^10,11^ However, the same OM structures of *P. putida* that are beneficial in its natural scenarios turn out to be an annoyance when the same bacteria are used in a variety of biotechnological settings.^12^ EPS production is not only energetically and metabolically costly, but it also causes biofouling in bioreactors.^13,14^ The same is true for flagella, the synthesis, turnover, and motion of which drains a good deal of cellular resources that could otherwise be directed to production of added-value molecules.^15^ In order to mitigate such drawbacks, variants of the archetypal *P. putida* strain KT2440 have been generated over the years that lack flagella,^15^ detrimental genomic parasites, and burdensome genomic segments.^16^ Not surprisingly, the resulting strains display a better tolerance to stress and a higher capacity for hosting heterologous genes and pathways.^17^ Further elimination of a large number of envelope structures (*i.e.*, pili, fimbria, surface proteins, exopolysaccharides) has resulted in *P. putida* variants with a smoother and more exposed cell surface^18^—with two important consequences. One is that they fail to build biofilms, as virtually all molecular mediators of intercell adhesion and attachment to solid materials have been eliminated. The other is that the exterior of such surface-naked bacteria have a much more accessible overlay that facilitates display and functionality of genetically encoded adhesins.^18^ These properties raise the opportunity to altogether reprogram the passive tropism of *P. putida* (*i.e.*, preferential attachment to a target object, whether biotic or abiotic) and thus create distinctive bacterial coatings on specific materials. Note that catalytic biofilms of this biotechnological platform have already been developed by submitting its endogenous cdGMP regulon to an external control.^19,20^ In contrast, what we entertain here is a complete replacement of the biofilm-forming native program of *P. putida* by an altogether synthetic surface-attachment machinery.

With these notions in mind, we set out to redesign such a tropism of *Pseudomonas putida* KT2440 and its surface naked variant *P. putida* EM371.^18^ To this end, different aspects needed to be considered. First is about the adhesin(s) that enable strong attachment to a physical object that exposes in turn a recognizable molecular motif. In the work below, we have adopted nanobodies (V_HH_s) as facilitators of the pursued interactions with objects overlaid with cognate antigens. V_HH_s are the variable regions of single-domain antibodies of camelids which are composed of just one polypeptide (∼15 kDa) while maintaining full recognition of their target epitopes.^21^ Next is the bacterial surface display system of the V_HH_ of choice, for which a number of molecular tools have been proposed, including for *P. putida*.^22−27^ In particular, modified autotransporter proteins have been developed as a means to expose proteins on the bacterial surface^28−31^ of a suite of Gram-negative bacteria.^32^ As shown below, the option in this case was merging the V_HH_ sequence to an intimin carrier. These are virulence-associated adhesins of certain pathogenic *E. coli* strains^33^ that have been instrumental before for outward-facing presentation of nanobodies in *E. coli*.^34^ Finally, for the sake of robust applications, a reliable measure of the strength of the bacteria-surface interactions needs to be put in place. In this work, the precise attachment metrics of bacteria onto surfaces was determined with a Quartz Crystal Microbalance (QCM).^35^ The technology involves an extremely sensitive mass detector that measures *in situ* changes in mass per unit area in the nanogram per cm^2^ range. This is because the frequency at which a piezoelectric quartz crystal resonates depends on the mass deposited on the surface as indicated in the Sauerbrey’s equation.^36^ This method allows continuous measurements in label-free systems and also provides kinetic information on the process and the resulting surface coverage as well.^37,38^ Simultaneously, samples can be visualized by scanning electron microscopy (SEM) and atomic force microscopy (AFM).^35,39^

By building on the techniques and biological components just listed, the work below reports the engineering of monolayers of *P. putida* cells ectopically expressing an antifibrinogen nanobody as synthetic adhesins toward a solid surface coated with the antigen. These results showcase how the endogenous surface-attachment program of environmental bacteria can be entirely supplanted by a rationally designed counterpart that follows human-designed inputs rather than naturally occurring cues. Furthermore, the data presented provide a proof of concept on how goods combining biotic/live materials with abiotic partners can be easily assembled for a suite of applications such as the engineering of living and smart materials with genetically programmable properties.^40,41^

## Results and Discussion

### Assembling an Intimin-Nanobody Fusion in *Pseudomonas putida*

**Figure 1:**
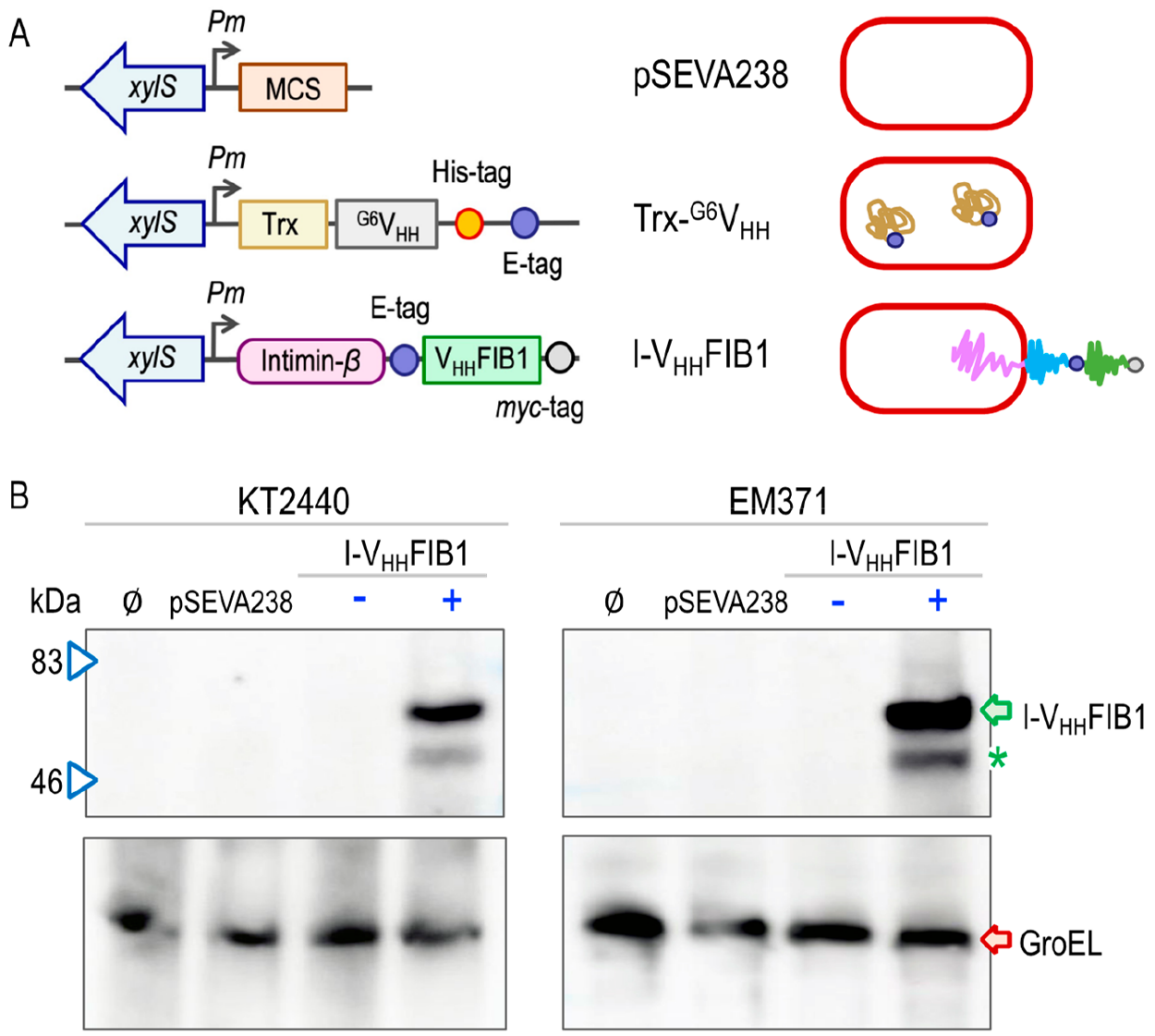
Constructs and expression of the intimin nanobody fusion in *P. putida* strains. (A) schematic representation of the constructs used to verify the proper surface display in *P. putida*. The β-domain of the intimin gene (purple) is fused to the V_HH_FIB1 nanobody (green). The nanobody DNA is flanked by both an E-tag (blue circle) and a *myc*-tag (gray circle) epitopes. The expression of this hybrid construct was placed under control of the XylS/*Pm* expression system of pSEVA238. As a cytoplasmic expression control, we used a construction cloned into the same expression pSEVA238 plasmid containing the thioredoxin domain (Trx) fused to the ^G6^V_HH_ nanobody followed by the His-tag and E-tag epitopes.^18^ We also used the pSEVA238 empty plasmid as negative expression control. Pictures are not drawn to scale. The left side part of the image shows the predicted bacterial configurations depending on whether it expresses either the empty plasmid (pSEVA238), the intracellular Trx-^G6^V_HH_ nanobody, or the surface displayed-nanobody (I-V_HH_FIB1). Pictures are not drawn to scale. (B) Western blot of *P. putida* KT2440 and EM371 GFP-labeled variants grown in LB medium in the absence or in the presence of inducer. Exponentially growing bacterial cells (OD_600_ of ∼03–0.5) were induced with 1 mM 3MBz and incubated at 30 °C for 3 h (OD_600_ ∼ 1.7). 10 μL of whole-cell protein extracts corresponding to identical cell numbers were loaded onto 10% (w/v) SDS-PAGE. KT2440 samples are on the left part and EM371 on the right. Cells without plasmid (⌀), with the empty plasmid (pSEVA238), with the recombinant plasmid (pSEVA238-I-V_HH_FIB1), without induction (−) or induced (+). The intimin nanobody recombinant protein was detected using an anti-E-tag-POD (upper images), while the heat shock protein GroEL was revealed with an anti-GroEL-POD (lower pictures). The mass of protein standards (kDa) is shown on the left part.

As explained above, nanobodies—the variable regions (V_HH_s) of single-domain antibodies of camelids^42^ and sharks^43^—look like a good choice as synthetic adhesins. While these molecules retain the full functionality of cognate antigen recognition, they are monomeric polypeptides of small size (∼15 kDa; cognate DNA ∼400 bp) with a good solubility and high physicochemical stability.^21^ Moreover, nanobodies can be easily generated to specifically target a desired interaction partner whether from libraries raised from naïve^44^ or immunized camels^21,45,46^ as well as from synthetic combinatorial pools.^47^ More recently ssDNA recombineering^48,49^ has expanded the choices for isolating nanobodies aimed at given purposes. In the case study reported below, we picked a well-characterized nanobody (V_FIB1_^50^) that recognizes human fibrinogen, a large and abundant glycoprotein, as its cognate antigen and which was isolated out of a display library in *E. coli*.^50^ In order to display such a nanobody on the surface of *P. putida*, the 2.4 kb XbaI-HindIII DNA insert of plasmid pNVfib1^50^ encoding a fusion between V_FIB1_ and the β-barrel of intimin was recloned into broad host range expression vector pSEVA238 to produce pSEVA238-I-V_HH_FIB1 (Supplementary Table S1) in which the hybrid, named hereafter I-V_HH_FIB1 (Figure 1A), is expressed under the control of the 3-methylbenzoate (3MBz)-inducible XylS/*Pm* system.^51^ The first 659 amino acids of the hybrid protein include the signal peptide (SP), the peptidoglycan binding motif (LysM), the β-barrel outer membrane anchoring structure and the Ig-like D0 domain^34^ of the naturally occurring intimin.^52,53^ This is followed by the V_FIB1_ nanobody sequence, that then occurs as a C-terminal addition to the intimin^50,54^ (Figure 1A). The V_FIB1_ DNA segment is flanked by E-tag and *myc*-tag epitopes to monitor expression and proper display of the thereby assembled recombinant adhesin on the surface of *P. putida*. The accuracy of the pSEVA238-I-V_HH_FIB1 architecture was first verified by PCR with oligos described in Supplementary Table S2 and then corroborated by DNA sequencing.

### Inducible Expression and Surface Display of V_HH_FIB in *P. putida*

The thereby generated plasmid pSEVA238-I-V_HH_FIB1 was passed to *P. putida* strains KT2440 (wild type), its surface-naked variant EM371, and their GFP-labeled derivatives^18^ (the last ones were the specimens used in the experiments discussed below). As a control, the same strains were transformed with insertless vector pSEVA238 (Figure 1A). To test bulk expression of the plasmid cargo, the transformants were grown with or without inducer and the extracts loaded in a SDS-PAGE followed by a western blot revealed with an anti-E-tag antibody. As shown in Figure 1B, samples from bacteria bearing the plasmid with the nanobody insert expressed the I-V_HH_FIB1 protein when added with 3MBz whether the host cells were *P. putida* KT2440 or *P. putida* EM371. Typically, induced samples produced a major band with an apparent size close to the predicted mass, along with a minor product (Figure 1B). However, such second band virtually disappeared when samples were pretreated with urea (Supplementary Figure S1). This suggested that the second band stemmed from the partial unfolding of the recombinant hybrid upon treatment with SDS and not from any instability or proteolytic degradation (such a resistance of β-barrels to denaturation has been reported before^55^). In order to test the gross physiological impact of I-V_HH_FIB1 expression, samples were also probed with an anti-GroEL antibody as a general stress reporter.^56,57^ As shown in the lower part of Figure 1B, the intracellular levels of this chaperone remained basically constant, indicating that production of the nanobody-intimin fusion was not utterly detrimental to the *P. putida* hosts.

**Figure 2:**
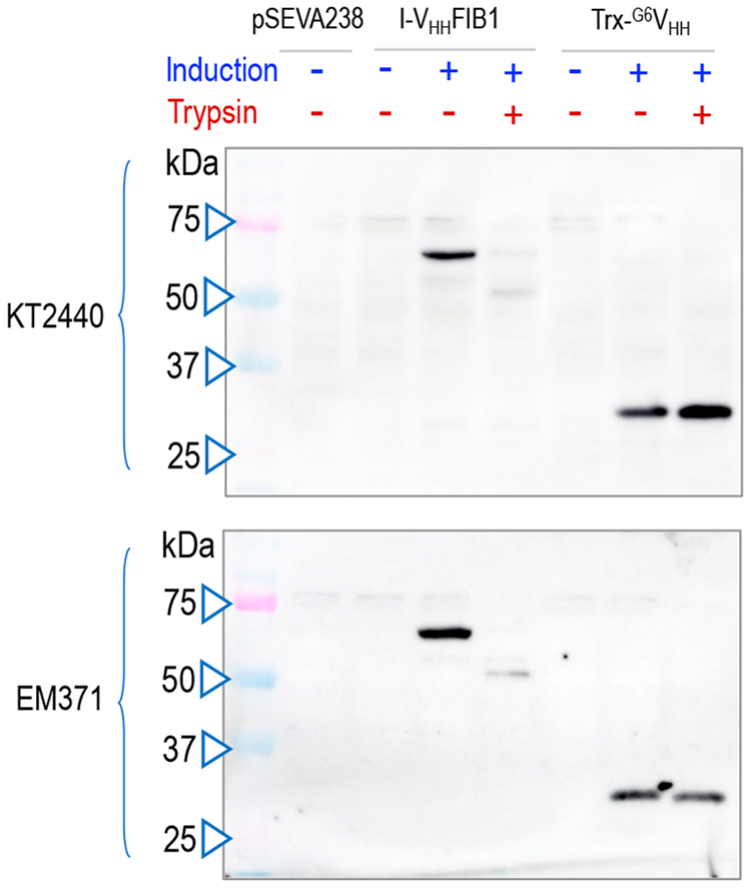
Protease accessibility assay to detect surface display of the recombinant adhesin in *P. putida* strains. Western blot of whole cell protein extracts of *P. putida* KT2440 or EM371 GFP-labeled strains with the empty vector (pSEVA238), with the recombinant adhesin (I- V_HH_FIB1), or with a thioredoxin domain fused to a nanobody (Trx-^G6^V_HH_). Moreover, cells were either induced (+) or not (−) with 1 mM 3MBz. Also, cells were treated (+) or not (−) with trypsin before protein extraction to eliminate surface exposed structures. The membrane was revealed with anti-E-tag as the primary antibody and this was detected with an appropriate secondary antibody conjugated with peroxidase. The mass of protein markers (kDa) is shown on the left part.

The next step was to investigate whether the intimin nanobody fusion was displayed on the surface of *P. putida*. For this, apart from the strains indicated we transformed the same variants with pSEVA238-trx-^G6^V_HH_ (Supplementary Table S1) as a protein localization control. As sketched Figure 1A, this construct expresses an intracellular nanobody (Trx-^G6^V_HH_) labeled with His and E-tag epitopes that remains inside the bacterial cytoplasm.^58^ In a first series of experiments, *P. putida* strains KT2440 and EM371 bearing pSEVA238, pSEVA238-trx-^G6^V_HH_, or pSEVA238-I-V_HH_FIB1 were grown as before, induced or not with 3MBz, and treated with a nonlethal concentration of trypsin. Since the protease cannot enter the cells, the rationale of this test is that proteins displayed on the bacterial surface are cleared off upon exposure to trypsin, while those located within the cytoplasm are due to stay intact.^18,59^Figure 2 shows a Western blot of proteins extracts from either *P. putida* KT2440 or *P. putida* EM371 with the different constructs and growth conditions, treated (+) or not (−) with trypsin. The data showed that the recombinant adhesin was indeed exposed on the surface of either strain, as the E-tag reactive band disappeared after treatment with the protease. In contrast, the intracellular nanobody control (Trx-^G6^V_HH_) was not affected by exposure to trypsin. As was the case with the expression data of Figure 1B, a residual fraction of the I-V_HH_FIB fusion remained unaffected by protease treatment (Figure 2 and Supplementary Figure S1), perhaps reflecting an intermediate step of the *in vivo* secretion route when the E-tag is not yet entirely exposed on the cell exterior.^34,55^ In any case, the data indicated a good level of expression and display of the V_HH_FIB nanobody that permitted moving to the next step.

### Monitoring Dynamics of Mass Attachment to Surfaces with QCM

**Figure 3:**
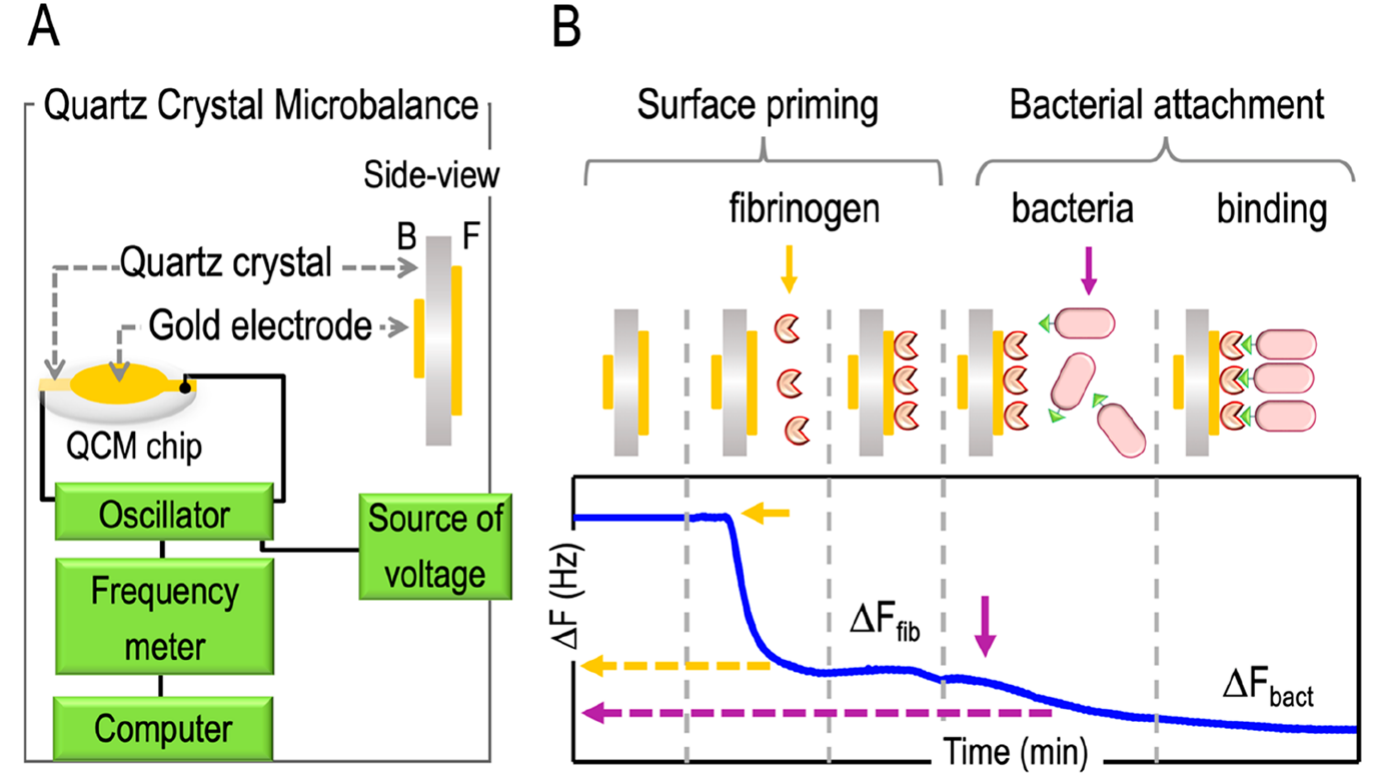
A Quartz Crystal Microbalance (QCM) apparatus and its use to detect bacterial attachment to a modified surface. (A) Schematic representation of the elements that constitute a QCM machine. (B) Experimental setup to monitor bacterial attachment to a fibrinogen coated surface. To do that, first a solution of fibrinogen (yellow arrow) is injected in the QCM and the changes in frequency monitored (Δ*F*_fib_) to confirm that the electrode has been correctly coated. Then, cells producing the recombinant adhesin are injected (purple arrow) into the QCM and frequency changes observed (Δ*F*_bact_) to detect bacterial attachment.

In order to test and quantify direct attachment of modified *P. putida* strains to a solid material a quartz crystal microbalance (QCM) device was adopted. This is an extremely sensitive mass sensor that provides information on particle immobilization events in real time in the nanogram range along with attachment/detachment rate constants.^35^ QCM measurements are based on the piezoelectric nature of quartz crystal, which can be made to oscillate at defined frequencies by applying an appropriate voltage. A schematic representation of the QCM apparatus is represented in Figure 3A. Addition or removal of small amounts of mass onto the electrode surface changes the oscillation frequency of the crystal (Δ*F*), which can be deconvoluted into kinetic data on the molecular interactions taking place at the electrode surface.^60^ In our case, the QCM platform was used for monitoring quantitatively the physical contacts between bacterial cells displaying the V_HH_FIB nanobody and a surface coated with its target antigen (fibrinogen). The QCM technology delivers a better performance to this end, as—unlike other methods, *e.g.*, ELISA^61^ —it measures the actual cell weight deposited on the surface, not just the strength of nanobody-antigen recognition.

For the actual experiments, the gold surface of the electrode was first primed with fibrinogen. To this end a flow of 1× PBS was injected into the system until the oscillation frequency stabilized (Figure 3B). Next, a 10 μg mL^–1^ fibrinogen solution was injected and changes in oscillation frequencies recorded (Figure 3B; Δ*F*_fib_). Supplementary Figure S2A shows that Δ*F* along time decreases once fibrinogen was injected. This was indicative of its binding to the gold surface of the quartz crystal through electrostatic and hydrophobic interactions.^62^ The mass of the immobilized protein can be estimated using Sauerbrey’s equation (see eq 1 in the Material and Methods section), assuming that the decrease of frequency observed is only due to the adsorption of the fibrinogen to the surface. In addition, assuming that the immobilization process is kinetically controlled the resulting frequency–time curve can be fit (Supplementary Figure S1A, red line) to a first-order kinetic equation (see eq 2 in the Material and Methods section). In that case, the Δ*F*_max_ and the kinetic constant (*k*) values were −70 Hz (±10) and 0.35 min^–1^, respectively, and the mass (Δ*m*) of immobilized fibrinogen onto the gold surface was estimated to be 1.2 × 10^3^ ng cm^–2^. Considering a fibrinogen molecular mass of 340 kDa, the surface coverage value was 3.53 × 10^–12^ mol cm^–2^. The theoretical monolayer coverage mass for fibrinogen, assuming long axis perpendicular to the surface (front size surface area 1.37 cm^2^), is 1.59 × 10^–12^ mol cm^–2^ (540 ng cm^–2^) calculated by a Random Sequential Adsorption (RSA) model.^63^ Since the value calculated by us is twice the theoretical value, we can conclude that the gold surface is completely covered by two monolayers of fibrinogen. Supplementary Figure S2 shows scanning electron microscopy images of the gold surface area of the QCM crystal before (Supplementary Figure S2B) and after the injection of the protein at stake (Supplementary Figure S2C). Compared with the image of the bare gold surface, after fibrinogen deposition a shiny granular deposit can be observed, confirming the complete coating of the surface with the protein.

### Binding *P. putida* Cells to a Fibrinogen-Layered Surface

**Figure 4:**
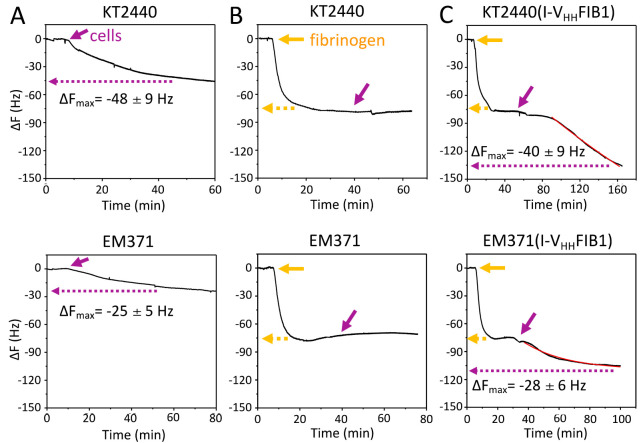
Functional validation of the exposed artificial adhesin in *P. putida* using a QCM. (A) Time dependence of frequency changes upon injection of cells, either KT2440 (upper part) or EM371 (lower), on the gold surface of the electrode. The addition of cells is depicted by a purple arrow and the Δ*F*_max_ represented within a dashed purple line within charts. (B) Time dependence of frequency changes upon injection of 10 μg mL^–1^ fibrinogen (yellow line) to the bare gold surface, and once the frequency stabilized (dashed yellow line) either KT2440 (upper) or EM371 (lower) cells were introduced into the system (purple arrow). (C) Frequency changes along time resulting from initial injection of 10 μg mL^–1^ fibrinogen (yellow line), and once the frequency stabilized (dashed yellow line) either KT2440 with or EM371 equipped with the artificial adhesin (I-V_HH_FIB1) introduced into the system. The dashed purple line indicates the Δ*F*_max_ and the red line corresponds to the fitting of the experimental data to a first-order kinetic equation. Note the difference in scale among different experimental conditions. These experiments were performed in 3 replicates: a representative experiment is shown.

On the basis of the above, attachment of bacteria to the thereby fashioned surface could be followed as the increase in bound mass reflected by changes in oscillation frequency (Δ*F*_bact_) of the quartz crystal that results from cells adhered to the immobilized antigen (Figure 3B). In order to generate blank references for the process the affinity of plasmidless *P. putida* KT2440 and *P. putida* EM371 cells toward the bare gold surface of the QCM crystal was tested. To do that, a diluted solution of cells (OD_600_ ∼ 0.3) in 1× PBS was injected into the system and the response of the electrode monitored along time. Figure 4A shows the changes in frequency observed in the wild type strain (Δ*F*_max_ −48 Hz ± 9) and in the naked variant (Δ*F*_max_ −25 Hz ± 5). Note that *P. putida* EM371 attached to the noncoated surface worse than *P. putida* KT2440, thereby highlighting the inability of the *naked* cells to stick to solid materials once deprived of OM structures.^18^ A second control involved testing attachment the same plasmidless *P. putida* KT2440 and *P. putida* EM371 cells to fibrinogen-coated electrode. For this, the system was first flown with a 10 μg mL^–1^ fibrinogen solution as before. When the oscillation frequency stabilized, samples of each strains diluted to an OD_600_ ∼ 0.3 in 1× PBS were then injected into the QCM apparatus and the response along time recorded (Figure 4B). As shown in Figure 4B, no change in the oscillation frequencies was detected upon addition of the nonrecombinant strains in either case.

Finally, we examined the attachment of the same *P. putida* hosts (whether KT2440 or EM371) transformed with plasmid pSEVA238-I-V_HH_FIB1 induced with 3MBz and thus expressing/displaying the encoded nanobody. As before, the QCM flow was first added with a fibrinogen solution followed by injection of the bacteria diluted in 1× PBS to OD_600_ ∼ 0.3. Figure 4C shows that a conspicuous change in Δ*F* was observed following inclusion of the cells regardless of the host strain. This was indicative of a specific interplay between the cellular bodies displaying the antifibrinogen nanobody and the antigen-coated surface. In the case of the wild-type *P. putida* KT2440 expressing I-V_HH_FIB1 the decrease in frequency was 40 Hz, which came down to 28 Hz when the host was the naked strain *P. putida* EM371. The frequency-time curves obtained for these strains were fit to a first-order kinetics equation (indicated by a red line within the plots of Figure 4C). On one hand, the resulting kinetic values (*k*) for attachment of either strain (0.003 min^–1^ for the wild type strain and 0.03 min^–1^ and the EM371 variant, respectively) indicated that mutual recognition between the nanobody-displaying cells and their cognate target was faster for the naked cells. This did not come as a surprise, as the lack of bulky surface structures in the naked strain^18^ should facilitate more immediate access of the cell-exposed nanobody to the immobilized fibrinogen. On the other hand, the Δ*F*_max_ figures obtained from the recombinant strains were −40 Hz ± 9 and 0.003 min^–1^ for *P. putida* KT2440 and −28 Hz ± 6 and 0.03 min^–1^ for *P. putida* EM371 (note the different time scale used in Figure 4C for each strain). By using Sauerbrey’s equation (see eq 1 in the Material and Methods section) we could then calculate the net bacterial biomass immobilized on the antigen-coated surface. On the basis of such Δ*F* changes, the mass gained upon bacterial attachment turned out to be 0.70 μg cm^–2^ for *P. putida* KT2440 (I-V_HH_FIB1^+^) and 0.44 μg cm^–2^ for *P. putida* EM371 (I-V_HH_FIB1^+^). Assuming that the weight of one bacterium is ∼650 × 10^–15^ g,^64,65^ the calculated number of cells deposited on the surface of the electrode was estimated to be ∼1 × 10^6^ bacteria cm^–2^ for *P. putida* KT2440 and ∼0.7 × 10^6^ bacteria cm^–2^ for the *P. putida* EM371 strain. These figures were somewhat paradoxical, as we expected a larger share of *P. putida* EM371 (I-V_HH_FIB1^+^) cells attached to the electrode surface. These data prompted us to inspect directly the type of physical association between the cells and the antigen-coated material with different microscopy techniques as explained below.

### Direct Visualization of *P. putida* Adhered to Antigen-Coated Materials

**Figure 5:**
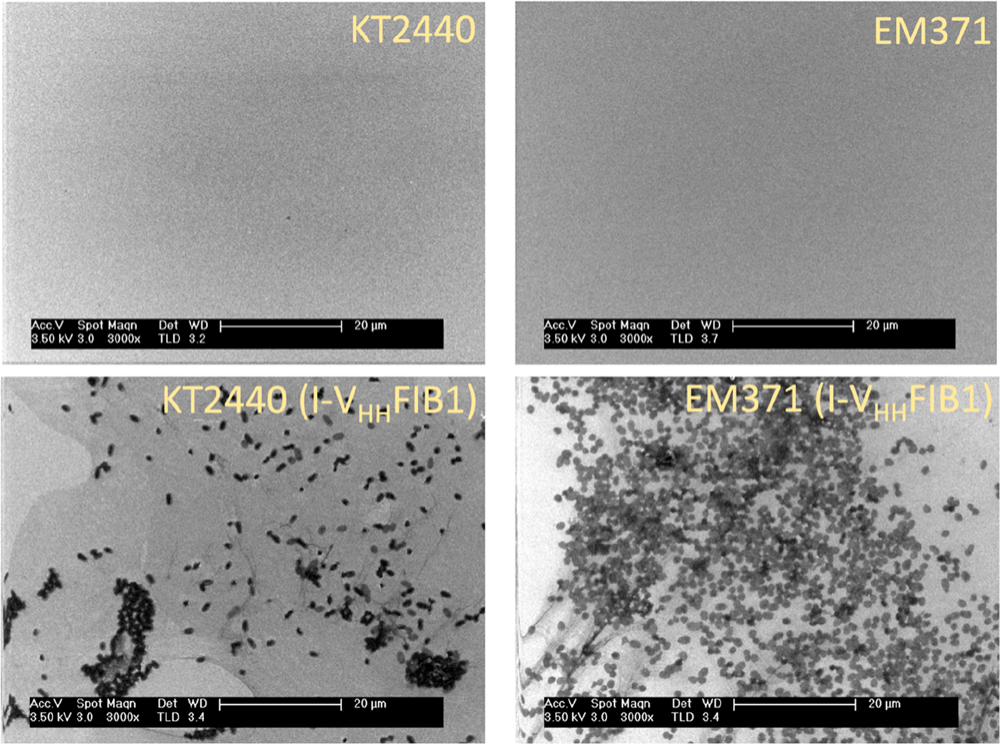
Scanning electron images (SEM) of the fibrinogen modified gold substrate after injection of plasmidless control strains (*P. putida* KT2440 and *naked* EM371) and cells expressing the recombinant adhesin, *P. putida* KT2440 (I-V_HH_FIB1) and *P. putida* EM371 (I-V_HH_FIB1) as indicated.

**Figure 6:**
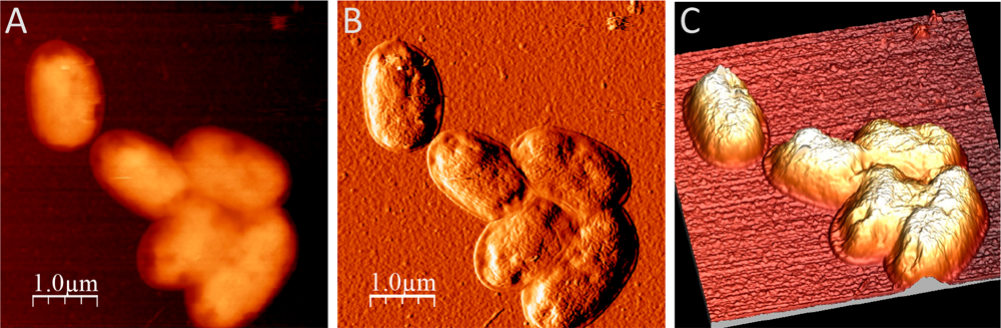
Topographic (A), phase (B), and 3D (C) AFM images of *P. putida* cells equipped with the recombinant adhesin performed in air using contact mode. Images were taken of *P. putida* KT2440 (pSEVA238-I-V_HH_FIB1) cells displaying nanobody I-V_HH_FIB1^+^ and stuck to the fibrinogen-layered electrode. Samples were subject to AFM as indicated in the text to inspect the appearance of the bacteria.

In order to gain some details on the interplay between the nanobody-presenting cells and the target surface, the electrodes used for the QCM experiments above were processed as indicated in the Materials and Methods and used to image the surface of the QCM crystal by scanning electron microscopy (SEM). The results shown in Figure 5 clarify the enigmatic data mentioned in the previous section on the values of capture of the biomass of wild-type and the surface-naked strain—both presenting the antifibrinogen nanobody. The top of Figure 5 shows the fibrinogen-coated QCM crystals following exposure to plasmidless *P. putida* KT2440 and *P. putida* EM371 cells. In both cases, virtually no bacteria could be found attached to the antigen-layered surface, thereby verifying the results reported in Figure 4B. However, when QCM crystals were analyzed with SEM after injecting the strains displaying I-V_HH_FIB1, cells attached onto the surface became clearly visible (Figure 5, bottom). Yet, the distribution of the cells on the plane differed significantly. While the biomass of the nanobody-presenting naked strain *P. putida* EM371 (I-V_HH_FIB1^+^) was evenly distributed as a monolayer of individual cells, that of the wild-type counterpart tended to form microaggregates in which lateral cell-to-cell contacts appeared to predominate in respect to those of discrete cells with the electrode. Some changes in cell morphology were also seen: wild type bacteria appeared more elongated and were intermingled with extracellular threads (possibly fimbriae and other EPS), while naked cells were rounder and more attached to the surface by themselves (Supplementary Figure S3). A plausible explanation for the higher net binding of bacterial biomass of *P. putida* KT2440 (I-V_HH_FIB1^+^) in respect to the naked-surface alternative is that they reflect two types of association. In one case (wild-type strain), the nanobody-mediated attachment is less efficient but suffices to create a nucleation site for further buildup of a microcolony kept together by naturally occurring means of intercell bonding and biofilm formation.^2,66,67^ In contrast, the surface-naked cells (which lack such ordinary course of sticking to neighbors^18^) are bound to the antigen-covered crystal exclusively through the artificial adhesins, thereby producing a *bona fide* bacterial monolayer spanning the whole surface. To further inspect the mode of binding of *P. putida* KT2440 (I-V_HH_FIB1^+^) to the fibrinogen-layered electrode, the SEM images shown in Figure 5 and Supplementary Figure S3, the same samples were subject to Atomic Force Microscopy (AFM) to morphologically analyze the aspect of the cells once bound to the surface. Figure 6 shows a representative AFM capture of *P. putida* KT2440 cells forcibly attached to such a plane. Despite the alterations caused by dehydration of the cells.^39^, bacteria could be sized with an average length of 1.8 ± 0.2 μm and 1.1 ± 0.1 μm width, respectively. That cells were strongly fastened to the surface is suggested by their flattened shapes surrounded by what appears to be a 70 nm corona of crushed membranes in intimate contact with the electrode. While these observations have limited quantitative value, they are compatible with a strong interaction with the solid layer. Similar AFM images were also obtained for *P. putida* EM371 displaying the recombinant adhesin and attached to a fibrinogen-coated surface (Supplementary Figure S4).

## Conclusion

*P. putida* is a nonpathogenic environmental bacterium^6^ that naturally thrives in soil and the plant rhizosphere, but has emerged also in recent years as a metabolic engineering platform for a suite of industrial and environmental applications.^10,11,68,69^ One promising stratagem to strengthen the biotechnological possibilities of such platforms is to combine the intrinsic catalytic abilities of the cells with their physical shapes and material properties in what has been called synthetic morphologies. Different approaches include altering cell shape,^70^ assembling synthetic consortia with a given architecture,^71,72^ and promoting adhesion to solid surfaces for forming catalytic biofilms.^73−75^ In the last case, the predominant strategy is the manipulation of the native cdGMP network of species/strain at stake for production of surface-gluing polymers that secure bacterial binding to any material at hand.^19,20^ In this work we present a further step in this direction by either (i) combining the natural biofilm forming potential of *P. putida* with an artificial device to provide a site of early attachment to a target solid—which facilitates later buildup of a standard biofilms—or (ii) replacing altogether the extracellular mediators of surface attachment by a synthetic one. In either case, the key instrument to this end is ectopic expression of a nanobody that recognizes a distinct, well-defined target, thereby acting as a synthetic adhesin.^54^ When such a device is expressed in wild-type *P. putida*, attachment of a few cells to the antigen-coated surface elicits formation of microcolonies on a material to which the nanobody-less bacteria is altogether unable to colonize (Figure 5; upper part). In this instance, the synthetic adhesin just promotes the necessary early attachment event that is followed by massive formation of a biofilm on a surface that cannot be otherwise colonized. In the second scenario (ectopic expression of a nanobody on an otherwise OM-naked cell), the result of the same operation is formation of a monolayer of cells strongly adhered to the antigen-coated solid. Note that—unlike the biofilm scenario—further growth of such monolayered cells does not thicken the adhered biomass: new cells either diffuse away or stick to not yet occupied antigen-coated surfaces.

Although the work described above was made at a laboratory scale and for the sake of validating the concept, we argue that its scale-up with other adhesins and different targets can open new avenues for the biotechnological exploitation of *P. putida*, *e.g.*, by combining its biological activities with other qualities provided by the nonbiological carrier—as pursued *inter alia* in the design of new functional, genetically programmable materials.^40,41^

## Materials and Methods

### Bacterial Strains, Plasmids, Culture Media, and Growth Conditions

The bacterial strains and plasmids used in this work are described in Supplementary Table S1. Cells were routinely grown in LB (10 g l^–1^ tryptone, 5 g L^–1^ yeast extract, and 5 g L^–1^ NaCl) and when required in M9 minimal medium supplemented with 0.2% (w/v) citrate as carbon source. *E. coli* cells were incubated at 37 °C while *P. putida* at 30 °C. Antibiotics were used at the following final concentrations: 50 μg mL^–1^ kanamycin (Km) and 30 μg mL^–1^ chloramphenicol (Cm). Proteins of interest were expressed with 1 mM 3-methylbenzoate (3MBz) at OD ∼ 0.3–0.5 for either 3 h or overnight.

### General DNA Techniques

DNA was manipulated using common laboratory techniques described in.^76^ Plasmid DNA was prepared using the QIAprep Spin Miniprep kit (Qiagen, Inc., Valencia, CA) and DNA was purified using the NucleoSpin Extract II (Macherey-Nagel, Düren, Germany). The oligonucleotides used in this work are listed in Supplementary Table S2. Colony PCR was done by picking cells with a sterile toothpick directly from agar plates into PCR reaction tubes.^77^

### Construction of Plasmids for Intimin-Based Surface Display in *P. putida*

Plasmid pNVfib1 harboring a truncated version of the intimin gene (*eae*) containing the β-domain fused to a nanobody (V_FIB1_) that targets human fibrinogen was obtained from ref (50). This construct (pNVfib1) was digested with XbaI/HindIII and the ∼2.4 kb fragment cloned first in pVLT35^78^ to yield -pVLT35-Nv. This plasmid was then digested with XbaI/HindIII and the ∼2.4 kb ligated into pSEVA238^79^ to obtain pSEVA238-I-V_FIB1_. The cargo region of the plasmid was sequenced using the oligos described in Supplementary Table S2. Plasmid constructs were introduced as indicated in each case into different *P. putida* strains either by electroporation or by conjugal transfer as described in the literature.^80^

### Protein Extracts, SDS-PAGE, and Western Blots

Whole cell protein extracts were prepared by harvesting 1 mL of induced bacteria (OD_600_ ∼ 1.5), resuspended in 50 μL of 10 mM TrisHCl pH 8.0 and mixed with 50 μL of 2× SDS-sample buffer (60 mM Tris-HCl pH 6.8, 1% (w/v) SDS, 5% (v/v) glycerol, 0.005% (w/v) bromophenol blue, and 1% (v/v) 2-mercaptoethanol) or 2× urea-SDS-sample buffer (60 mM Tris-HCl pH 6.8, 2% (w/v) SDS, 4 M urea, 5 mM EDTA, 5% (v/v) glycerol, 0.005% (w/v) bromophenol blue and 1% (v/v) 2-mercaptoethanol) as described in the literature.^55^ Then, samples were boiled for 15 min and sonicated for 5 s (Labsonic B Braun) to completely disrupt cells. Next, cell debris and insoluble material were eliminated by centrifugation at 14 000*g* for 5 min. Proteins were analyzed by loading onto 10% (w/v) SDS-PAGE gels and resolved with a Miniprotean III electrophoresis system (Bio-Rad). For western blot, proteins were transferred from SDS-PAGE gels to a polyvinylidene difluoride membranes (PVDF, Inmobilon-P, Merck Millipore, MA, USA) using a Trans-Blot SD semidry transfer cell (Bio-Rad; CA, USA). Then, membranes were blocked in phosphate buffered saline (PBS; 8 mM Na_2_HPO_4_, 1.5 mM KH_2_PO_4_, 3 mM KCl, 137 mM NaCl, pH 7.0) with 3% (w/v) skimmed milk (Milk-PBS) for 1 h (h) at room temperature (RT). Next, membranes were incubated for 1 h at RT in the same buffer (milk-PBS) with a 1/2000 dilution of the monoclonal anti-E-tag (Phadia, Sweden) antibody. Then, membranes were washed three times with milk-PBS buffer containing 0.1% (v/v) Tween-20 to eliminate unbound antibodies. After that, a 1/5000 dilution of antimouse IgG conjugated with peroxidase (POD; Merck, MO, USA) was used to locate the anti-E-tag bound protein. Finally, membranes were soaked into BM Chemiluminescence Blotting Substrate (POD; Merck, MO, USA) and incubated for 1 min in the dark and the PVDF-membranes were scanned in an Amersham Imager 600 (GE Healthcare, IL, USA).

### Protease Accessibility Assay

One milliliter of a culture of bacteria harboring the constructs indicated was induced with 1 mM 3MBz at OD ∼ 0.3–0.5 and let grow up to an OD_600_ ∼ 1.5, at which point cells were harvested by centrifugation at 4000*g* for 3 min and resuspended in 100 μL of 10 mM Tris HCl pH 8.0. This suspension was incubated with 200 μg mL^–1^ trypsin for 20 min at 37 °C. Next 5 μg mL^–1^ trypsin inhibitor (trypsin-chymotrypsin inhibitor; Sigma-Aldrich) was added to stop further proteolysis. Samples were then centrifuged at 14 000*g* for 1 min and the pellet resuspended in 50 μL of 10 mM Tris HCl pH 8.0 and processed for analysis in SDS-PAGE and western blot as indicated above.

### Quartz Crystal Microbalance Experiments

Quartz Crystal Microbalance (QCM) measurements were carried out using an SRS QCM200 model (SRS Instruments; Sunnyvale, CA, USA) equipped with wafer-shaped 5 MHz AT-cut quartz crystals of 25.4 mm of diameter and 331 μm thickness with circular gold electrodes deposited over a chromium adhesion layer. The circular electrode has an asymmetric configuration with a working area in the front side, facing the solution, of 1.37 cm^2^, while the piezoelectric area of the backside was 0.40 cm^2^. Before each measurement crystals were cleaned in a 5:1:1 solution of milli-Q water, 35% (v/v) H_2_O_2_ and 25% (v/v) NH_3_ at 75 °C for 5 min. After that, crystals were washed with water and dried. The quartz crystal resonator was placed in a Teflon probe and vertically immersed in thermostatted water-jacketed beaker at 30 °C for measurements. First, the system was stabilized with PBS buffer carrier using a constant flow of 0.04 mL min^–1^. Then, a 10 μg mL^–1^ fibrinogen solution was injected and the frequency was allowed to reach a steady state, indicating that the gold surface of the quartz crystal is covered by fibrinogen. After that, the bacterial samples diluted in PBS at an OD_600_ of 0.3 samples were injected, and changes in frequency along time was monitored until the system reached a steady state. Frequency changes are related to the mass changes at the electrode surface by the Sauerbrey’s equation:^36^
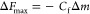
1where Δ*F*_max_ is the frequency change in hertz (Hz), *C*_f_ is the sensitivity factor for the crystal used (56.6 Hz μg^–1^ cm^–2^ for a 5 MHz AT-cut quartz crystal at room temperature), and Δ*m* is the mass change (μg cm^–2^). Strictly, this equation is good for systems in air and for mass additions forming an evenly rigid layer on the active sensor area.^81^ Nevertheless, it is widely accepted to estimate the adsorbed mass in liquid environments.^36,82−84^ Assuming that the immobilization process is kinetically controlled, the frequency–time curves can be fit to a first-order kinetics equation in order to determine the kinetic constant (*k*):
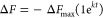
2where Δ*F* is the frequency changes in hertz, Δ*F*_max_ is the frequency change between the initial and the stabilized state, *k* is the first-order kinetic constant (min^–1^), and *t* is the time.

### Scanning Electron (SEM) and Atomic Force Microscopy (AFM)

Experiments for SEM and AFM were done on QCM crystals modified either with fibrinogen or with fibrinogen and the bacterial strains indicated in each case in a PBS carrier under constant flow of 0.04 mL min^–1^. Samples were visualized with an ultrahigh-resolution scanning electron microscope Philips XL30 S-FEG. In the case of AFM, all morphology measurements were performed in air at room temperature (25 °C) using an Agilent 5500 microscope operating in contact mode. Olympus cantilevers (RC800PSA, 200_20 mm) with a tip radius of *ca*. 20 nm and spring constants of 0.15_0.6 N/m were used.

## Supporting Information Available

Supplementary Table S1 (Bacterial strains and plasmids used in this work); Supplementary Table S2 (Oligonucleotides used in this study); Supplementary Figure S1 (Expression of the recombinant adhesin in of *P. putida* KT2440 and EM371); Supplementary Figure S2 (QCM electrode surface preparation); Supplementary Figure S3 (SEM images of the fibrinogen-coated surface after injection with KT2440 (I-V_HH_FIB1) or EM371 (I-V_HH_FIB1); Supplementary Figure S4 (Topographic, phase, and 3D AFM images of *P. putida* EM371 cells equipped with the recombinant adhesin, performed in air using contact mode) (PDF)
