# Supplementary material for: Engineering Tropism of Pseudomonas putida toward Target Surfaces through Ectopic Display of Recombinant Nanobodies
Source: ACS Synth Biol. 2021 Aug 2;10(8):2049–59. doi: 10.1021/acssynbio.1c00227 (PMC8397431; doi:10.1021/acssynbio.1c00227)
Supplement: Supplementary file 1 — sb1c00227_si_001.pdf [file sb1c00227_si_001.pdf]

SUPPLEMENTAL MATERIAL TO

Engineering tropism of *Pseudomonas putida* towards target surfaces through ectopic display of recombinant nanobodies

by

Sofía Fraile[1+], María Briones[2+], Mónica Revenga-Parra[2], Víctor de Lorenzo[1*], Encarnación Lorenzo[2] and Esteban Martínez-García[1]

[1]Systems Biology Department, Centro Nacional de Biotecnología (CNB-CSIC), [2]Departamento de Química Analítica y Análisis Instrumental; Institute for Advanced Research in Chemical Sciences (IAdChem), Universidad Autónoma de Madrid and IMDEA-Nanociencia, Campus de Cantoblanco, 28049 Madrid, Spain.

[+] Both authors contributed equally

Supplementary Table S1. Bacterial strains and plasmids used in this work.

Strain / plasmid	Relevant characteristics [a)]	Source
Escherichia coli		
CC118	Cloning host; Δ(*ara-leu*) *araD* Δ*lacX74 galE galK phoA thiE1 rpsE* (Sp^R) *rpoB* (Rif^R) *argE*(Am) *recA1*	[1]
HB101	Mating helper strain; F^- λ^- *hsdS20*(r_B^- m_B^-) *recA13 leuB6*(Am) *araC14* Δ(*gpt-proA*)62 *lacY1 galK2*(Oc) *xyl-5 mtl-1 thiE1 rpsL20*(Sm^R) g*lnX44*(AS)	[2]
Pseudomonas putida		
KT2440	Prototrophic, reference strain	[3]
EM371	KT2440 surface-naked derivative	[4]
KT2440-GFP	KT2440 labelled with GFP	[5]
EM371-GFP	EM371 labelled with GFP	[4]
Plasmids		
pRK600	Helper vector for mating; *oriV* (ColE1); RK2(*mob*$^+$ *tra*$^+$); Cm^R	[6]
pNVfib1	Expression plasmid; *oriV* (ColE1); l*acI*^{q}-P_{lac} controlling the expression of the intimin$_{EHEC}$-E-V_{FIB1}-myc fusion; Cm^R	[7]
pVLT35	Expression plasmid; *oriV* (RSF1010); l*acI*^{q}-P_{tac}; Sm/Sp^R	[8]
Nv-pVLT35	Expression plasmid; *oriV* (RSF1010); l*acI*^{q}-P_{tac} controlling the expression of the intimin$_{EHEC}$-E-V_{FIB1}-myc fusion; Sm/Sp^R	This work
pSEVA238	Expression plasmid; *oriV* (pBBR1); *xylS*-P_m; Km^R	[9]
pSEVA238-I-V_{FIB1}	Expression plasmid; *oriV* (pBBR1); *xylS*-P_m controlling the expression of the intimin$_{EHEC}$-E-V_{FIB1}-myc fusion; Km^R	This work

		4
pSEVA238-trx-$^{G6}V_{HH}$	Expression vector; oriV (pBBR1); xylS-P_m→ trx-$^{G6}V_{HH}$; Km^R	

Supplementary Table S2. Oligonucleotides used in this study.

Name	Sequence 5´→3´	Usage	Reference
PS1	AGGGCGGCGGATTTGTCC	To check correctness of recombinant plasmids	[10]
PS2	GCGGCAACCGAGCGTTC	To check correctness of recombinant plasmids	[10]
238F	GGTTTGATAGGGATAAGTCCAG	To check correctness of recombinant plasmids	[11]

Supplementary Figure 1. Expression of the recombinant adhesin in *P. putida* KT2440 and EM371 GFP-labelled variants.

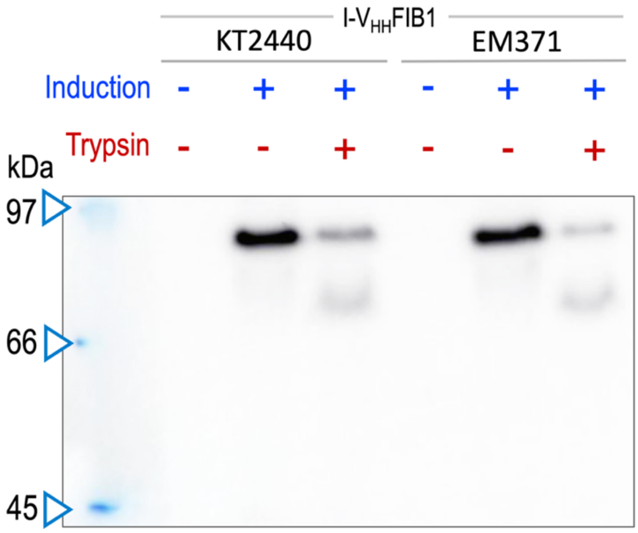

Western blot probed with anti-E-tag of whole cells extracts prepared in urea-SDS sample buffer of KT2440 and EM371 carrying the pSEVA238-I-V$_{HH}$FIB1 plasmid. Cells were induced with 1mM 3mBz and treated (+) or not (-) with trypsin. The molecular masses of the protein (KDa) standards are shown on the left.

Supplementary Figure 2. QCM electrode surface preparation.

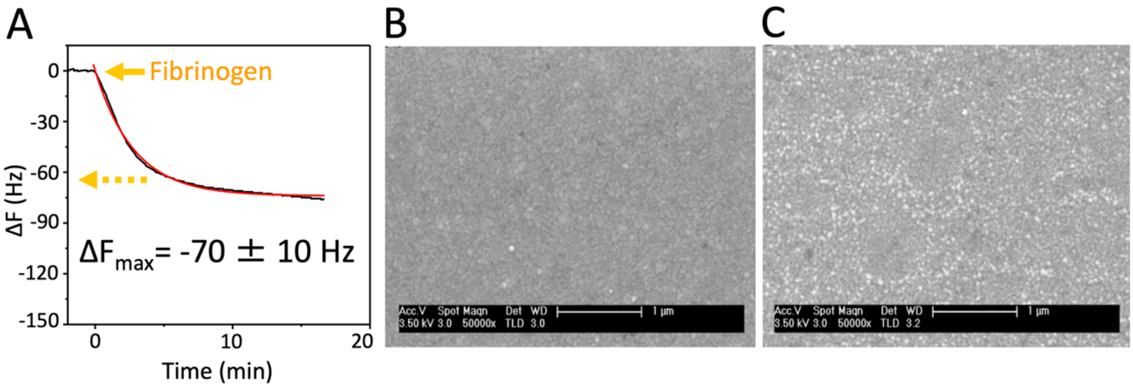

(A) Time dependence of the frequency changes of a bare gold substrate in PBS upon injection of 10 μg mL^{-1} fibrinogen solution. The red line corresponds to the fitting of the experimental data to a first-order kinetic equation. (B) Scanning electron microscopy image of the bare gold surface. (C) Scanning electron microscopy image of the electrode surface after adsorption of fibrinogen.

Supplementary Figure 3. SEM images of the fibrinogen-coated surface after injection with *P. putida* KT2440 (I-V_{HH}FIB1) or *P. putida* EM371 (I-V_{HH}FIB1).

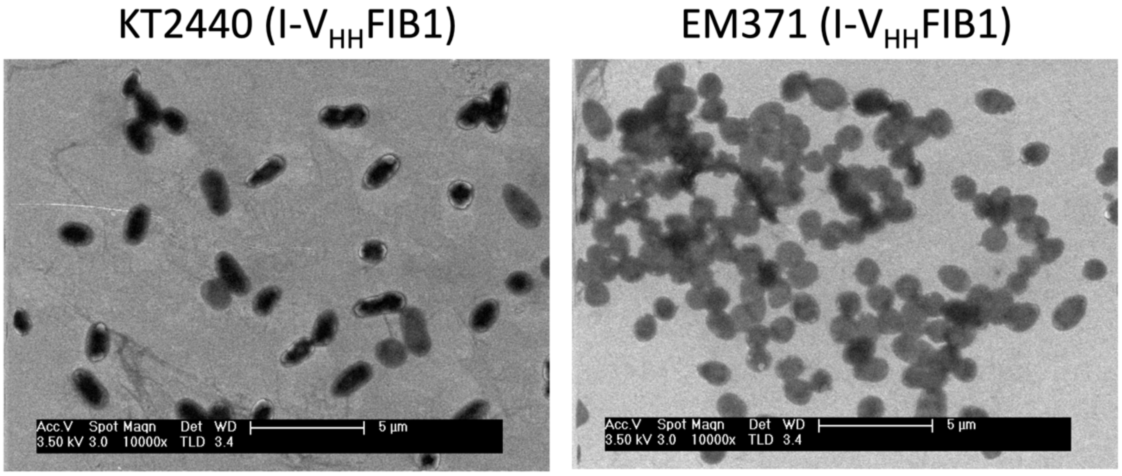

Images are higher-resolution pictures of the same samples shown in Fig. 5 of the main text.

Supplementary Figure 4. Topographic (A), phase (B) and 3D (C) AFM images of *P. putida* EM371 cells equipped with the recombinant adhesin performed in air using contact mode

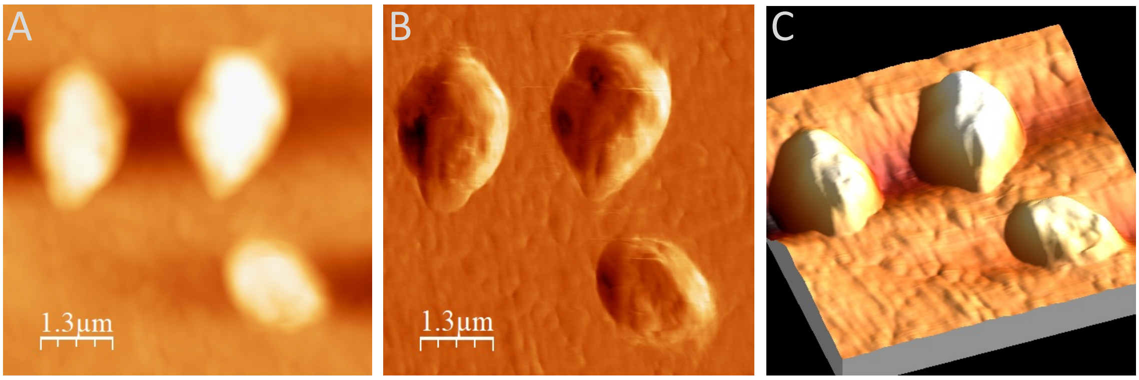

Images of *P. putida* EM371 (pSEVA238-I-V_{HH}FIB1) expressing the nanobody I-V_{HH}FIB1^{+} and attached to a fibrinogen coated gold surface.

REFERENCES

(1) Manoil, C., and Beckwith, J. (1985) TnphoA: a transposon probe for protein export signals. Proc Natl Acad Sci USA 82, 8129-8133.

(2) Boyer, H. W., and Roulland-Dussoix, D. (1969) A complementation analysis of the restriction and modification of DNA in *Escherichia coli.* J Mol Biol 41, 459-472.

(3) Bagdasarian, M., Lurz, R., Ruckert, B., Franklin, F. C., Bagdasarian, M. M., Frey, J., and Timmis, K. N. (1981) Specific-purpose plasmid cloning vectors. II. Broad host range, high copy number, RSF1010-derived vectors, and a host-vector system for gene cloning in *Pseudomonas*. Gene 16, 237-247.

(4) Martínez-García, E., Fraile, S., Rodríguez Espeso, D., Vecchietti, D., Bertoni, G., and de Lorenzo, V. (2020) Naked Bacterium: Emerging Properties of a Surfome-Streamlined *Pseudomonas putida* Strain. ACS Synth Biol 9, 2477-2492.

(5) Espeso, D. R., Martínez-García, E., de Lorenzo, V., and Goñi-Moreno, Á. (2016) Physical Forces Shape Group Identity of Swimming *Pseudomonas putida* Cells. Front Microbiol 7, 1437.

(6) Kessler, B., de Lorenzo, V., and Timmis, K. N. (1992) A general system to integrate *lacZ* fusions into the chromosomes of Gram-negative eubacteria: regulation of the Pm promoter of the TOL plasmid studied with all controlling elements in monocopy. Mol Gen Genet 233, 293-301.

(7) Salema, V., López-Guajardo, A., Gutiérrez, C., Mencía, M., and Fernández, L. A. (2016) Characterization of nanobodies binding human fibrinogen selected by *E. coli* display. J Biotechnol 234, 58-65.

(8) de Lorenzo, V., Eltis, L., Kessler, B., and Timmis, K. N. (1993) Analysis of *Pseudomonas* gene products using *lacI*^{q}/P*trp-lac* plasmids and transposons that confer conditional phenotypes. Gene 123, 17-24.

(9) Martinez-Garcia, E., Aparicio, T., Goni-Moreno, A., Fraile, S., and de Lorenzo, V. (2015) SEVA 2.0: an update of the Standard European Vector Architecture for de-/re-construction of bacterial functionalities. Nucleic Acids Res 43, D1183-1189.

(10) Silva-Rocha, R., Martínez-García, E., Calles, B., Chavarría, M., Arce-Rodríguez, A., de Las Heras, A., Páez-Espino, A. D., Durante-Rodríguez, G., Kim, J., Nikel, P. I., Platero, R., and de Lorenzo, V. (2012) The Standard European Vector Architecture (SEVA): a coherent platform for the analysis and deployment of complex prokaryotic phenotypes. Nucleic Acids Res 41, D666-675.

(11) Ricaurte, D. E., Martínez-García, E., Nyerges, Á., Pál, C., de Lorenzo, V., and Aparicio, T. (2018) A standardized workflow for surveying recombinases expands bacterial genome-editing capabilities. Microb Biotechnol 11, 176-188.
